# Variation of *BMP3* Contributes to Dog Breed Skull Diversity

**DOI:** 10.1371/journal.pgen.1002849

**Published:** 2012-08-02

**Authors:** Jeffrey J. Schoenebeck, Sarah A. Hutchinson, Alexandra Byers, Holly C. Beale, Blake Carrington, Daniel L. Faden, Maud Rimbault, Brennan Decker, Jeffrey M. Kidd, Raman Sood, Adam R. Boyko, John W. Fondon, Robert K. Wayne, Carlos D. Bustamante, Brian Ciruna, Elaine A. Ostrander

**Affiliations:** 1 Cancer Genetics Branch, National Human Genome Research Institute, Bethesda, Maryland, United States of America; 2 Program in Developmental and Stem Cell Biology, The Hospital for Sick Children, Toronto, Ontario, Canada; 3 Zebrafish Core, National Human Genome Research Institute, Bethesda, Maryland, United States of America; 4 Department of Genetics, Stanford School of Medicine, Stanford, California, United States of America; 5 Department of Biomedical Sciences, Cornell University College of Veterinary Medicine, Ithaca, New York, United States of America; 6 Department of Biology, University of Texas at Arlington, Arlington, Texas, United States of America; 7 Department of Ecology and Evolutionary Biology, University of California Los Angeles, Los Angeles, California, United States of America; 8 Department of Molecular Genetics, University of Toronto, Toronto, Ontario, Canada; University of Bern, Switzerland

## Abstract

Since the beginnings of domestication, the craniofacial architecture of the domestic dog has morphed and radiated to human whims. By beginning to define the genetic underpinnings of breed skull shapes, we can elucidate mechanisms of morphological diversification while presenting a framework for understanding human cephalic disorders. Using intrabreed association mapping with museum specimen measurements, we show that skull shape is regulated by at least five quantitative trait loci (QTLs). Our detailed analysis using whole-genome sequencing uncovers a missense mutation in *BMP3*. Validation studies in zebrafish show that Bmp3 function in cranial development is ancient. Our study reveals the causal variant for a canine QTL contributing to a major morphologic trait.

## Author Summary

As a result of selective breeding practices, modern dogs display a multitude of head shapes. Breeds such as the Pug and Bulldog popularize one of these morphologies, termed “brachycephaly.” A short, upward-pointing snout, a massive and rounded head, and an underbite typify brachycephalic breeds. Here, we have coupled the phenotypes collected from museum skulls with the genotypes collected from dogs and identified five regions of the dog genome that are associated with canine brachycephaly. Fine mapping at one of these regions revealed a causal mutation in the gene *BMP3*. Bmp3's role in regulating cranial development is evolutionarily ancient, as zebrafish require its function to generate a normal craniofacial morphology. Our data begin to expose the genetic mechanisms unknowingly employed by breeders to create and diversify the cranial shape of dogs.

## Introduction

Canine skull shape variation among dog breeds is in large part a human-created phenomenon, occurring through artificial selection and consolidation of desired traits. Morphological distinction between wolves and dogs dates as far back as 31,000 years ago [1], [2]. Changes in skull shape are a key feature of dog domestication, foreshadowing the wide variety of shapes displayed by modern dog breeds.

Skull shapes differ tremendously from one another, so much so that such differences are breed-defining. Two such skull shapes are brachycephaly (“shortened head”, e.g. Bulldog, Pug, Boxer) and dolichocephaly (“elongated head”, e.g. Greyhound, Saluki, Collie), which are named after their resemblance to human cephalic disorders. Although canine cranial shape is subject to multigenic control [3]–[5], the molecular underpinnings of this variation remain poorly defined. Candidate gene studies failed to uncover compelling causal variants of canine brachycephaly [6]–[8]. Airorhynchy (dorsal bending of the snout; a feature common to brachycephalic breeds) and midface length was previously correlated with polyglutamine and polyalanine repeat length of the transcription factor RUNX2 [4]. More recently, genome wide association scans (GWAS) and homozygosity mapping have converged on chromosome 1 (CFA1) as a locus that is highly associated with brachycephaly, implicating a 296 kb haplotype that spans *THSB2* and intergenic sequence proximal to *SMOC2* [3], [9], [10].

Here we present data indicating that at least five genetic loci are responsible for the cranioskeletal differences that differentiate dolichocephalic and brachycephalic dog breeds. Our conclusions are based on a GWAS that coupled craniometric breed-sex averages collected from 533 modern specimens from museum and private collections with the genetic profiles of 576 purebred dogs (62 breeds) assayed via single nucleotide polymorphism (SNP) chips. To identify candidates of phenotype causality, we filtered genetic variants derived from whole genome sequencing of eleven different breeds. This led to discovery of a compelling candidate for causality at the CFA32 QTL: a derived missense mutation in *BMP3* that is nearly fixed among small, brachycephalic dog breeds. To evaluate the functional potential of this variant *in vivo*, we turned to zebrafish. We show that Bmp3 is indispensable for normal craniofacial development in zebrafish, and comparison of missexpression assays using BMP3 and its canine variant suggests enhanced activity in the latter. Together, our data reveal for the first time the molecular underpinnings of a quantitative trait, selected by dog fanciers to modulate a prominent morphological trait in domestic dogs.

## Results

**Figure 1:**
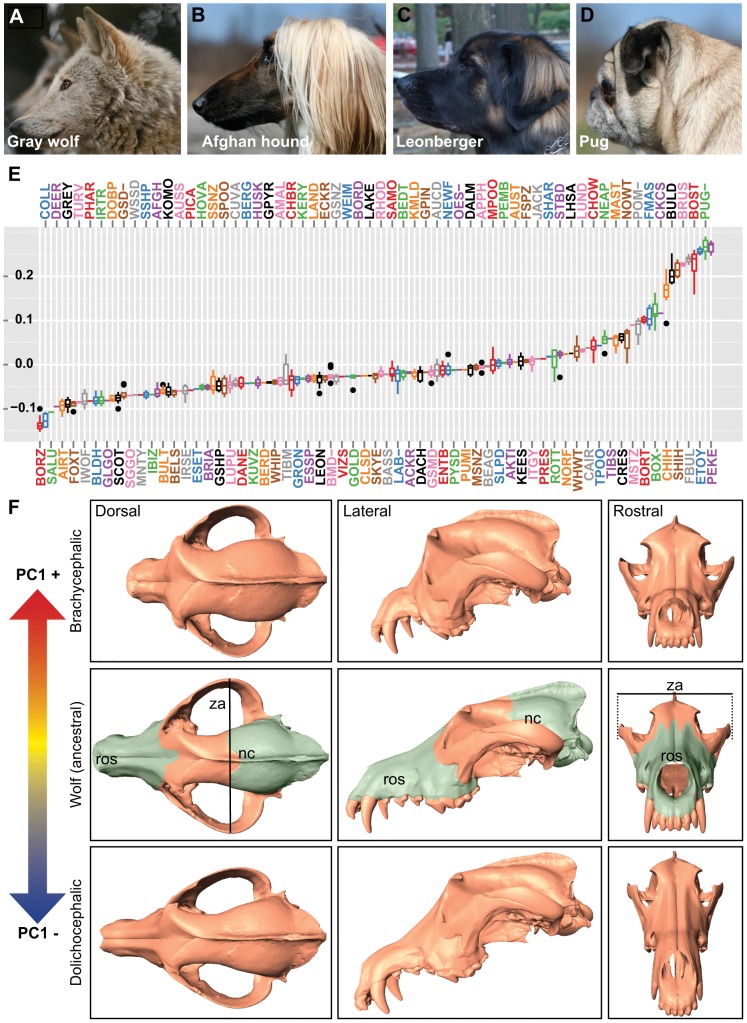
Quantitative and qualitative assessments of PC1 on canine cranioskeletal shape. (A) Gray wolf (mesocephalic, ancestor to dogs) (B) Afghan hound (dolichocephalic), (C) Leonberger (mesocephalic), (D) Pug (brachycephalic). (E) Boxplots of PC1 (corresponding breed names are listed in Table S2). (F) Surface scans of a gray wolf skull illustrate morphological changes associated with PC1. Columns (left to right) are dorsal, lateral, and rostral views. Top row: a gray wolf skull morphed by positive PC1. Middle row: a gray wolf skull (no morphing). Bottom row: a gray wolf skull morphed by negative PC1. Pseudocoloring of the gray wolf skull indicates rostrum (ros) and neurocranium (nc). Line indicates width of the zygomatic arches (za).

To capture the three-dimensional morphological complexities present among modern dogs, we digitized 51 stereotyped landmarks from 533 skulls representing 120 breeds and four gray wolf subspecies (Figure 1A–1D, Figure S1A–S1E, Tables S1 and S2). As most skulls used in our study originated from museums, we selected only those specimens with unambiguous breed designations, sex status, and recent time of death (within the past 40 years) for use in this study. Using MorphoJ software [11], we identified four principal components that accounted for nearly 75.5% of shape variance, with the majority of variance explained by the first component (PC1 = 59.4%, PC2 = 8.2%, PC3 = 4.2%, PC4 = 3.8%). PC1 describes profound changes in rostrum length and angle, palate and zygomatic arch width, and depth of the neurocranium: essentially the continuum of cranioskeletal features that extend between dolichocephalic and brachycephalic breeds (Figure 1E, 1F).

Since purebred dogs must conform to specific morphological standards [12], morphological traits like skull shape became highly uniform by breed, permitting association studies using one set of samples for genotyping and others for phenotyping. This strategy of using breed stereotypes has proven successful in mapping a number of canine morphologic traits by independent groups [3], [13], [14]. Using breed allele frequencies collected by the CanMap project [3], we conducted genome-wide scans of QTLs associated with breed-sex averages for PC1 (1–10 specimen(s)/breed/sex, mean n = 3, Table S3).

Initially, we scanned for PC1 associations using an additive linear regression model (Figure S2A, Table S4) [15]. Size correction in the regression suggested potential confounders (compare Figure S2A and S2B) on CFA10 and 15, which were previously associated with body size [3], [13], [14], [16]. As expected, addition of log(neurocranium centroid) breed-sex values as a covariate removed those associations (Figure S2B, see Materials and Methods for more details).

**Figure 2:**
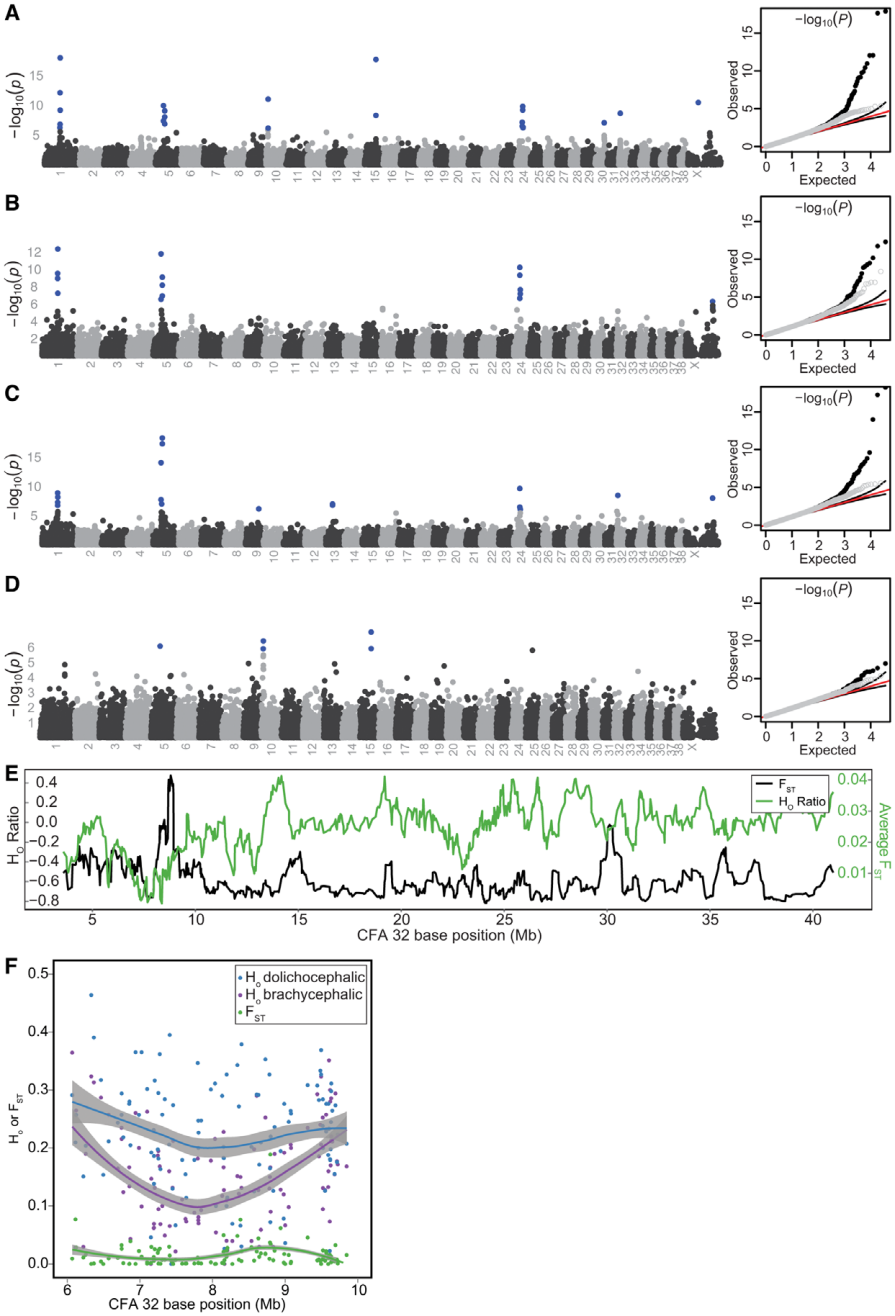
PC1 GWAS and fine mapping at CFA32. All GWAS used the mixed-model GEMMA. Chromosomes listed on the *x*-axis, −log_10_(*P*) on the *y*-axis. SNPs remaining significant following Bonferroni correction are colored blue. Q-Q plots of observed versus expected −log_10_(*P*) are depicted on right, with full SNP dataset (black circles), pruned dataset (grey circles), expected values (red lines), and 95% confidence intervals (black lines). Scan results using breed-sex averages of PC1 without (A) and with a breed-sex average size covariate (B). Including a size covariate in the mixed-model overcorrects, leading to loss of associations on CFA 30 and 32.(C) Scan results using PC1 breed-sex averages and breed-sex size covariates. In this scan, only breeds whose neurocranium size ranked within the smallest 50% of our dataset where analyzed. By reducing relatedness disparity in our study population, the association on CFA32 remains significant despite size correction. (D) Scan results using all breed-sex averages of PC1, but excluding extreme brachycephalic breeds (Pug, Pekingese, Boston Terrier, Shih Tzu, Brussels Griffon, French Bulldog, Bulldog, Boxer, Cavalier King Charles Spaniel, Chihuahua). (E) Average log(H_O_ ratios) or F_ST_ from ten-SNP sliding windows. (F) Regional H_O_ or F_ST_ values, and their respective Lowess best fit curves.

False associations derived from breed relatedness were excluded using GEMMA [17]. Discounting associations on CFA10 and 15, we identified six PC1-associated regions of interest indicated by SNPs at CFA1.59832965, CFA5.32359028, CFA24.26359293, CFA30.35656568, CFA32.8384767, and CFAX.44401786 (−log_10_(*P*) = 6.13–17.9, Figure 2A, Table S4). Of note, a suggestive association on CFAX was also observed, marked by SNP CFAX.104724717. Including a neurocranium centroid size covariate in the mixed-model removed associations at CFA10 and 15, as well as those on CFA30, 32, and X.44401786 and enhanced the association on CFAX.104724717 to significance (Figure 2B, Table S4). Since nearly all extreme brachycephalic breeds used in our study are also small breeds, and therefore substantially related to small, non-brachycephalic breeds, we reasoned that use of a size covariate in the mixed-model was overcorrecting associations that could be driven by diminutive breeds [3], [18], [19]. To reduce the contrast in relatedness among our study population, we reran the mixed-model using only breeds with a log(neurocranium centroid) below the 50^th^ percentile. This resulted in recovery of the CFA32 QTL, as well as new associations marked by SNPs at CFA9.50988217 and CFA13.26492600. Although the association on CFA30 remained below threshold for statistical significance, its association markedly improved (Figure 2C). When brachycephalic breeds were removed from the mixed-model, all aforementioned markers dropped below significance except for CFA5.36476657 (Figure 2D). Summarizing these findings, QTLs on CFA1, 5, 24, 32, and X (X:104724717) account for skull shape changes that occur along the continuum of canine brachycephaly-dolichocephaly. Additional associations reside on CFA9, CFA13, CFA30, and CFAX (X: 44401786), though their instability across mixed-model scans suggest they are either allometric in nature, driven by variation that is marginally represented by the breed composition present in our GWAS, or possibly false positives (Figure 2A–2D, Table S4).

Because shape variation is the result of artificial selection, we expected critical loci to be marked by reduction of observed heterozygosity (H_o_) and elevated genetic differentiation (F_ST_), hallmarks of selective sweeps [20], [21]. Among autosomes, QTLs on CFA1, 5, 30, and 32 displayed particularly strong reductions in H_O_ among brachycephalic breeds, relative to dolichocephalic breeds (H_R_, see Materials and Methods). Sliding H_R_ windows corresponding to these QTLs placed with the smallest <0.2% of the distribution. Among sliding window F_ST_ averages, windows corresponding to CFA1, 5, 24, 30, and 32 placed within the top 99.6% of the distribution (Figure 2E–2F, Figure S3A–S3I, and Table S5).

We focused on the CFA32 QTL because it was the second most highly associated, non-allometric locus in our initial analysis (Figure S2A), it showed compelling evidence of selection, and unlike the CFA1 QTL, it was previously unexplored [9]. Haplotype sharing at this locus among six of the seven extreme brachycephalic breeds, including the French Bulldog, Bulldog, Boston Terrier, Pekingese, Pug, and Brussels Griffon defined a critical interval spanning 190 kb (8,152,258–8,342,370, Table S6). Although this region included just two genes, both were excellent candidates: cGMP-dependent protein kinase 2 (*PRKG2*) and bone morphogenetic protein 3 (*BMP3*) [22]–[27].

**Figure 3:**
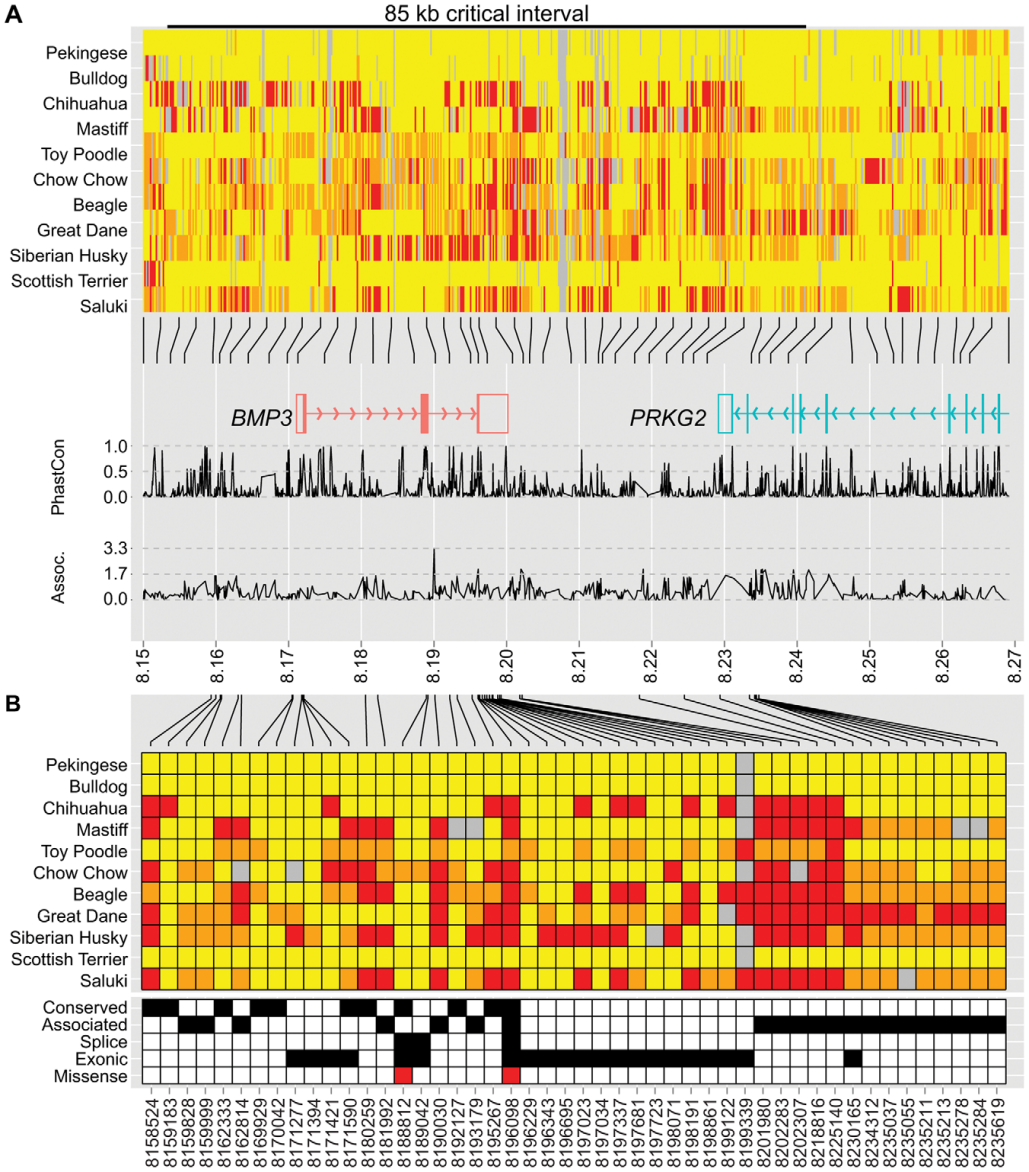
Genetic variation at the CFA32 QTL includes a brachycephaly-associated missense mutation within *BMP3*. For display purposes, we set the reference sequence to be the allele most common to Pekingese and Bulldog. Variants located within 8.15–8.27 Mb (A) or the 85 kb critical interval (B) are illustrated (homozygous reference = yellow, heterozygous = orange, homozygous variant = red). (A) Pekingese and Bulldog agree across an 85 kb interval (black bar) including *BMP3* (red) and a portion of *PRKG2* (aqua). Line graphs below genes plot conservation (phastCons4way) and association (−log_10_(*P*)) with respect to variant position (*28*). (B) Variants of interest met one or more of the following criteria: conserved (phastCons4way score ≥0.7), associated (an association *P*-value among the smallest 5% of *P*-values, see Materials and Methods), exonic (untranslated regions and coding), or splice (located within 20 bp of an exon boundary). Forty-eight variants of interest remained after applying filtering criteria, including a F452L mutation in *BMP3* at position 8,196,098.

To identify variants within the critical interval, we used whole-genome sequence analysis from eleven dog breeds of widely varying skull shapes (unpublished data). Notably, brachycephalic breeds including a Pekingese and a Bulldog were among the eleven, enabling the evaluation of phenotype association with genotype at nearly every position. Initial examination of variant calls in the 190 kb critical interval revealed over 2,000 polymorphisms (Table S7). Of particular interest, allelic differences between the Bulldog and Pekingese extended downstream of 8,237,936, suggesting a recombination breakpoint in the Pekingese. Confirmation of this breakpoint among 25 additional Pekingese reduced the critical interval to 85 kb (8,152,258–8,237,937 kb) (Figure 3A, Table S8).

Within the 85 kb interval, 48 variants that met one or more standard criteria were retained for further evaluation (Figure 3B). Only one variant remained a compelling candidate for causality: a SNP at 8,196,098 that encodes a missense mutation in BMP3, changing a phenylalanine to a leucine (*BMP3^F452L^* or F452L). The Protein Specific Scoring Matrix (PSSM) for TGF-β superfamily members indicates that position 452 is nearly invariably occupied by an aromatic amino acid such as tyrosine or phenylalanine (PSSM raw frequency = 0.84) and PolyPhen-2 substitution modeling predicted that the F452L substitution is likely damaging (HumDiv = 1.0, HumVar = 0.97) [28]. Moreover, F452 flanks highly conserved residues predicted to reside at the receptor-ligand interface [29]. Finally, expanded genotyping among 842 dogs from 113 breeds revealed that the *BMP3^F452L^* mutation is nearly fixed among extreme brachycephalic breeds. Furthermore, the PC1 scores of most carrier breeds fall between wolves (ancestral) and extreme brachycephalic breeds (Table S9).

**Figure 4:**
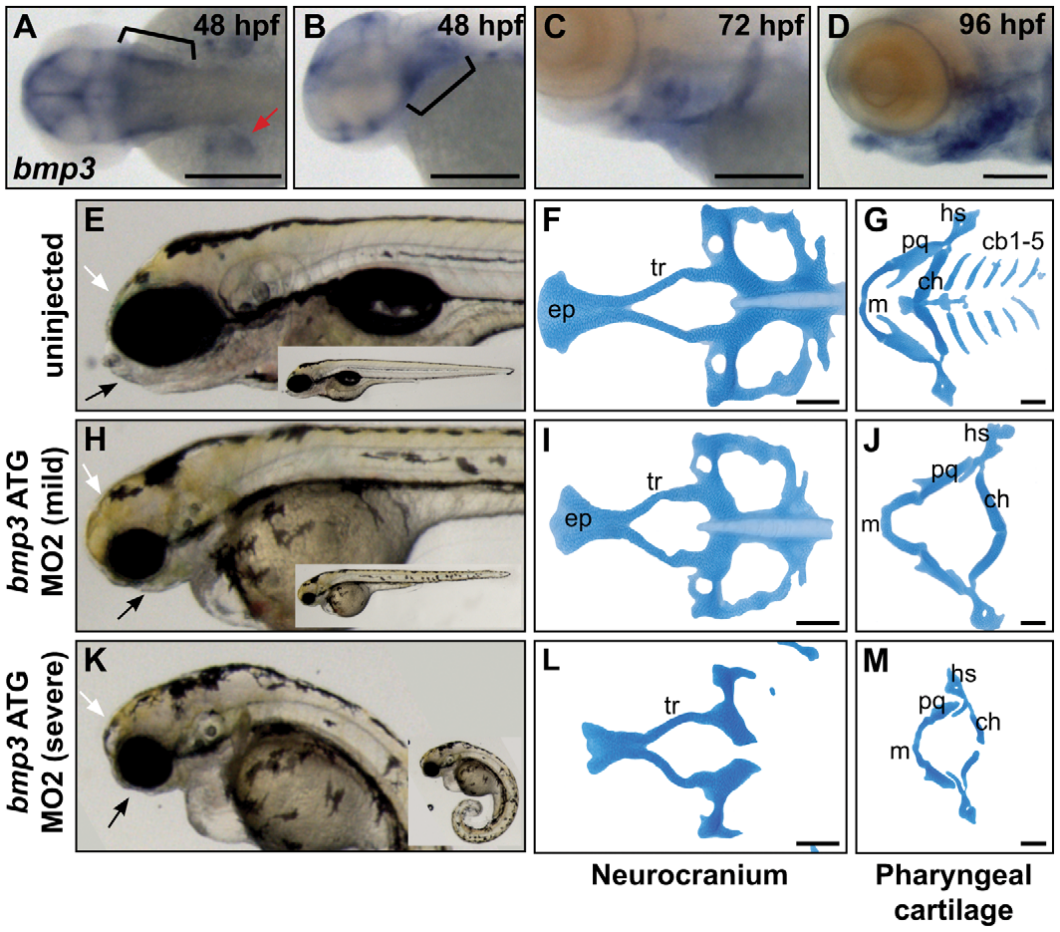
Zebrafish cranioskeletal development requires Bmp3 function. (A–H) Wholemount RNA *in situ* hybridization of *bmp3* expression at 48 hpf (A,B), 72 hpf (C), and 96 hpf (D) stages. Anterior to the left. (A) Dorsal view, (B–D) lateral view. Pharyngeal arches indicated by brackets, pectoral fins by red arrowheads. Wholemounts (E,H,K) and alcian blue cartilage stains (F,G,I,J,L,M) of 96 hpf embryos from uninjected (E–G) and morpholino-injected embryos (h–j, mild phenotype, n = 72/177; k–m, severe phenotype, n = 83/177). Phenotypic severity is distinguished by tail curling (compare insets). Loss of jaw structures (black arrows) and frontal bossing (white arrowheads) is apparent in both classes of morphants. Cartilage is severely dysmorphic, hypoplastic, or absent following Bmp3 knockdown. Abbreviations correspond to ceratobranchial (cb), ceratohyal (ch), eythmoid plate (ep), hyosymplectic (hs), Meckel's (m), palatoquadrate (pq), and trabeculae (tr) cartilages.

BMP3's role in cranioskeletal development is enigmatic in terms of molecular interactions and function. BMP3 antagonizes other BMPs and Activins through binding the ActRIIb receptor, and *in vivo*, BMP3 appears to restrict bone growth [23], [30], [31]. However, the absence of a knockout mouse craniofacial phenotype suggested that BMP3 function might be subtle, dispensable, or divergent to other mammals. We therefore assayed BMP3 function using the zebrafish model. Based on peptide similarity and synteny to CFA32 (96.4% identical within mature protein, 60.5% overall), the *BMP3* ortholog was identified on zebrafish chromosome 5. Endogenous expression of zebrafish *bmp3* is highly dynamic, first appearing during mid-somitogenesis as ubiquitous expression throughout the head, brain ventricles, and as was shown previously, the posterior somites (data not shown) [32]. After 48 hours post fertilization (hpf), *bmp3* expression emerges in pectoral fins, the pharyngeal arch region, heart, and jaw structures (Figure 4A–4D, data not shown). Prechondrogenic expression of *bmp3* among cranial structures suggests a role for Bmp3 in cranioskeletal development. To formally test this hypothesis, we knocked down endogenous Bmp3 activity via injection of translation-blocking antisense morpholino oligonucleotides (MO). Strikingly, MO-injected embryos demonstrated severe deficiencies in jaw development (Figure 4E, 4H, 4K). Alcian blue staining revealed loss or hypoplasia of multiple cartilage elements that form the viscerocranium and neurocranium (Figure 4F, 4G, 4I, 4J, 4L, 4M). Cartilage defects are specific to loss of Bmp3 activity since injection of two non-overlapping MOs produced identical craniofacial phenotypes, as did co-injection of both MOs at concentrations insufficient to cause phenotypes when injected alone (data not shown). These results indicate that Bmp3 is required for zebrafish craniofacial development, and indicate that Bmp3's role in craniofacial development is ancient. Furthermore, overexpression assays using *BMP3*, as well as other TGFβs, indicate that variation at the F452L residue has context-dependent effects on these molecules' activities (Figures S4, S5).

## Discussion

Distortion of the skull, as observed among brachycephalic and dolichocephalic dog breeds, affects bones presumably derived from endochondral and intramembraneous ossification. We show that the genetic basis of this distortion is complex, relying on the contributions of at least five QTLs. We propose that the *BMP3^F452L^* variant was selected by dog fanciers for its influence on skull shape, but the specific aspects of cranioskeletal development that the F452L variant affects within the brachycephalic skull remain unclear.

Previous studies, as well as ours, indicate that the CFA1 QTL is highly associated with canine brachycephaly and is robust to size-stratified GWAS (Figure 2A and 2C, data not shown), suggesting that the underlying causal variant at this locus is shared by both large and small brachycephalic breeds [3], [9]. Homozygosity mapping also implicated selective sweeps on CFA1, as well as CFA26, among Boxers, French Bulldogs, and Bulldogs [10]. Despite different morphometric approaches, skulls specimens, and utilization of CanMap genotypes profiles, our QTLs overlap with those reported by Boyko *et al.* for snout length (CFA1, 5, 32, X), cranial vault depth (CFAX), palate width (CFA30), and zygomatic arch width (CFA24) [3]. The associations that we report on CFA9 and 13 were revealed following size-stratified scans, raising caution regarding the implementation of mixed-model scans among domesticated populations whose traits and relatedness are difficult to disentangle. Notably, a snout ratio QTL on CFA9 was previously reported by Jones et al. in a study that also used breed stereotypes as phenotypes; our data independently replicates their finding [14].

We chose the zebrafish model to validate our GWAS results based on its rapid development, gene conservation, and flexibility for rapidly knocking down and overexpressing gene products of interest. Though loss-of-function using zebrafish indicates an ancient role for Bmp3 during craniofacial development, ontogenetic differences between teleost and amniote cranial development limit the extent to which specific phenotypic features can be recapitulated in both zebrafish and dogs.

*Bmp3* ^−/−^ mice described by Daluiski et al. have excessive trabeculation of the long bones, but defects in the cranial bones were not reported [23]. Interestingly, when authors of this study moved the *Bmp3* null allele to an inbred background, *Bmp3*−/− mutants died perinatally due to lung defects. A preliminary craniofacial analysis of E18.5 embryos suggests that a number of morphogenesis defects occur in the mutants (unpublished data, JJS and KLM; personal communication with KLM). In dogs, the *BMP3* mutation is but one of at least five QTLs that modulate canine skull shape variation. Thus, it is possible that genetic interactions with other QTLs enhance or act permissively to BMP3^F452L^'s effects on cranioskeletal development.

Microdeletions that include or flank *BMP3* are described in humans [33]. Although craniofacial abnormalities associated with these microdeletions were attributed to loss of *PRKG2*, our results suggest that haploinsufficiency for *BMP3* might also contribute to the clinical features of 4q21 syndrome. Furthermore, isolated BMP3 dysfunction could be the basis of human cephalic conditions whose genetic etiologies remain unknown.

The development of modern dog breeds is one of the most extensive genetic experiments ever conducted. Their existence allows us to exploit breed-average phenotypes for genetic analysis. In the past, the extensive linkage disequilibrium inherent to artificial selection often hindered the process of fine mapping causal variants in the dog [16]. We overcame this limitation using whole-genome sequencing to comprehensively evaluate candidate variants. Combining the resulting insights with the functional utility of zebrafish, we identified a causal mutation underlying a quantitative trait in the dog. Together these approaches have allowed us to extend the paradigm of leveraging breed-average phenotypes to include the identification of causal mutations. We can now work towards assembling the full inventory of genes associated with vertebrate cranioskeletal shape, in turn illuminating evolutionarily conserved mechanisms of cranioskeletal development in our own species.

## Materials and Methods

### Morphometrics and CanMap phenotype assignments

Fifty-one measurements were captured using an Immersion MicroScribe Digitizer G2X running Microscribe Utility Software and Diagnostics (v5.0.0.2). In total, 533 canid skulls representative of 120 breeds and 4 gray wolf subspecies located in museums and private collections were documented. Dorsal and ventral landmark datasets were captured separately and merged based on landmarks in common between datasets (landmarks 1, 2, 28, and 29) using File Converter software (Klingenberg lab). Procrustes fit, PCA, and residuals were generated using MorphoJ [11]. Residuals of nonallometric shape were calculated as implemented in MorphoJ (v1.03a) using linear regression (pooled by sex and breed), with symmetric component and log(neurocranium centroid) corresponding to dependent and independent variables, respectively. Ten thousand permutations were performed. Refer to Figure S1 to see landmarks used by MorphoJ to calculate neurocranium centroid.

A covariance matrix based on residuals was analyzed by PCA. GWAS was performed using a subset of the CanMap dataset of genotypes [3]. In total, 72 breed-sex averages of PC1 were assigned to CanMap breeds. In 30 instances, only one skull per breed-sex was measured. In such cases, the actual PC1 score was used for CanMap phenotype assignments. Log(neurocranium centroid) values were similarly assigned and used in subsequent analyses as a size covariate for PLINK and GEMMA association analyses (see next section).

Skull surface scans (1 Pug, 1 gray wolf) were done by Konica Minolta (3D Sensing Labs, Ramsey, NJ). Decimated scans were loaded into Landmark Editor (v3.6) [34]. Skull morphing was done using PC1 landmark coordinates exported from MorphoJ. Coordinate files used for morphing were generated from representatives of dolichocephalic and brachycephalic breeds (a Collie and Pug).

### Genomic analyses

Base pair positions stated throughout refer to CanFam2 (Broad/May 2005) coordinates. Single marker and haplotype association analyses were done using PLINK (v1.07) [15] or mixed model GEMMA (v0.91) [17] where specified. CanMap markers used in the analysis included SNPs with missingness <0.10 and minor allele frequency >0.01. In the full dataset (all breeds with breed-sex PC1 averages), 61,270 SNPs were analyzed by PLINK from 576 dogs representing 62 American Kennel Club-recognized breeds. In the mixed-model, ∼36,685 SNPs were analyzed. Breeds used in size-stratified analyses are listed in Table S2. Significantly associated SNPs surpassed Bonferroni correction at the 0.05 level (−log10(*P*)> = 5.86). H_O_ was calculated by treating CanMap breeds at the polar extremes of PC1 as two comparisons populations (Pug, Pekingese, Boston Terrier, Shih Tzu, Brussels Griffon, French Bulldog, Bulldog, Boxer, Cavalier King Charles Spaniel, Chihuahua versus Collie, Borzoi, Saluki, Scottish Deerhound, Bloodhound, Greyhound, Scottish Terrier, Doberman Pinscher, and Irish Wolfhound). F_ST_ was calculated treating brachycephalic breeds (listed above) as a single subpopulation. H_O_, H_R_ (the ratio of dolicho- and brachycephalic H_O_), and F_ST_ values were calculated using custom R scripts. fastPHASE was used to generate haplotype frequencies by breed, using CanMap genotypes using the clustering parameter k = 15 [35]. “Extreme brachycephalic breeds” were designated as such if both PC1 breed-sex averages exceeded 0.15. This cutoff was chosen based on the obvious jump in magnitude of PC1 values (see Figure 1E, Figure S6). Breeds that meet this classification include the Pug, Pekingese, Boston Terrier, French Bulldog, Bulldog, Brussels Griffon, and Shih Tzu.

### Sample collection, Sanger sequencing, and genotyping

DNA used in our study was extracted from blood samples as previously described [16]. In addition to whole-genome sequencing (see below), *BMP3* and *PRKG2* were Sanger sequenced using six brachycephalic and six dolichocephalic breeds (data not shown). The *BMP3* 8,196,098 C/A transversion was sequenced in an expanded panel composed of 847 dogs from 113 breeds. Primers were designed with a melting temperature (T_m_) ranging between 68–72°C, GC content ranging between 20–80%, length ranging between 18–32 nucleotides, and included 5′ M13 tags (Table S10). PCR products for sequencing were generated with a 2-step thermocycler program:

Initial Denaturation: 1×—95°C, 5 minutes
Two-step Cycles: 35×—95°C, 30 seconds; 68°C, 2 minutes
Extension: 1×—72°C, 10 minutes

PCR products were sequenced using a standard protocol [16]. During the course of SNP discovery, we discovered errors in the reference genome sequence for canine *BMP3*, producing two early stop codons in the first exon. Sequencing of 13 dogs, including the individual from which the reference genome sequence was derived, indicates these stop codons are the results of errors in the reference sequence.

### Whole-genome sequencing and variant filtering

Paired-end libraries were prepared from DNA from eleven dogs of breeds with widely varying skull shapes. Sequencing was conducted on an Illumina HiSeq 2000 sequencer to a depth of 5.6–8.5× per dog using manufacturer protocols. The resulting 101-base paired-end sequences were mapped to the genome (CanFam2 release May 2005) with bwa version 0.5.9-r16 with read trimming set to 15. SNPs were called with samtools mpileup version 0.1.18 and custom R scripts [36]–[39]. Thirteen SNPs in the PC1-associated region overlap with the CanineHD Genotyping BeadChip (Illumina cat. no. WG-440). DNA from four dogs was assayed with the chip; all resulting genotypes were identical in the deep sequencing and chip results. Four hundred and fifty-two SNPs were identified in the critical interval (85.7 kb between 8,152,258 and 8,237,937), and subjected to further filters. Genotypes with a genotype quality score below 8 were reset to “unknown.” We performed association analysis using PLINK with options specifying an additive model omitting the Scottish Terrier, a dolichocephalic breed that appears to be an outlier [15]. After correcting for multiple testing, no SNPs were significantly associated due to limited statistical strength of the test. SNPs in the 5th percentile for association scores were retained. Cross-species conservation was assessed by the UCSC phastCons4way calculations [40] downloaded November 30, 2011, which is generated by using the phastCons program to score the extent of conservation between dog, human, mouse and rat. SNPs with a phastCons4way score above 0.7 were retained. SNPs in an exon or within 20 bases of a splice junction were retained.

### Morpholino injections

Morpholino knockdown experiments of *bmp3* used two translation blockers (MO1: 5′-TGACAGCGATCCATGCTGGAGGTGC-3′, MO2 5′-CGGGACTATGGAAGCTGATCTA-3′), which overlapped by one nucleotide. Morpholino injections used 5.1 ng (MO1) or 7.5 ng (MO2), as determined by titrations.

### RNA synthesis and injections

Zebrafish *bmp3* (IMAGE Id 7052011) and human *BMP3* (Origene clone SC302990) cDNAs were sequenced and determined to be full length. Missense F→L mutations for mouse *GDF1* [41], human *Bmp3*, and zebrafish *bmp2b* [42] were introduced using site-directed mutagenesis and confirmed by sequencing. Zebrafish *bmp3* wt and F→L cDNAs were PCR-amplified using gene-specific primers with attB sites. PCR products were subcloned into entry and destination vectors (pCSDest) using Gateway recombination, as previously described [43], [44]. To construct the human *BMP3* expression vector, we PCR-amplified the TGF-β signaling domain using primers with XbaI and XhoI restriction sites. PCR products were ligated into an expression vector bearing the Xenopus *BMP2* prodomain, as such heterologous fusion constructs were previously shown to enhance propeptide cleavage and biological activity [41]. mRNA was synthesized using Ambion's SP6 mMessage kit from plasmid that was linearized with Not I. Embryo analyses of RNA injections were done based on injections of the following amounts: 25–300 pg human *BMP3* mRNA, 25–300 pg mouse *Gdf1* mRNA, 1–100 pg zebrafish *bmp2b*. mRNA overexpression assays were repeated three or more times at each stated concentration, unless stated otherwise.

### *In Situ* and Alcian blue stains

*In situ* hybridization was completed as described in Thisse and Thisse 2008 [45], except probes were hydrolyzed for 2 minutes at 65°C, the hybridization solution contained 5% dextran sulfate, and the anti-DIG-AP incubation and subsequent washes were performed in Malic Acid Buffer rather than PBST. Alcian blue stains were done as previously described by Schilling *et al.* [46], except that staining solution was composed of 0.15% Alcian blue, 50% EtOH, and 0.1 M HCl (pH = ∼1).

### Plots and images

Embryos were imaged using Zeiss Axio Imager.M1, Zeiss SteREO Lumar v12, or Leica M216F compound microscope. Zeiss Axiovision v4.8.1 software was used for image capture. Nonspecific background and dissection debris were removed from images of Alcian blue cartilage dissections using Adobe Photoshop CS3. All plots were generated using custom scripts, in conjunction with R Cran packages ggplot2 [38], reshape2 [39], and RColorbrewer [47]. Manhattan plot and Q-Q plot scripts were adapted from examples posted on the blog “Getting Genetics Done” [48]. Post-processing of plots was done using Adobe CS4 Creative Suite softwares Photoshop, InDesign, and Illustrator.

### Ethics statement

Informed consent was obtained for all collected dog samples. All animal protocols (dog and zebrafish) were approved by the Animal Care and Use Committees of the Intramural Program of the National Human Genome Research Institute at the National Institutes of Health or by Animal Care Committee of the Hospital for Sick Children Research Institute. Wild canids samples were graciously provided by Dr. Robert Wayne, in accordance with UCLA Approved Animal Care and Use Committee Policies.

## Supporting Information

Figure S1 Diagrams of 51 cranioskeletal landmarks captured with a microscribe digitizer. See Table S1 for anatomical descriptions corresponding to numbering. (a–e) Anterior facing left. (A) Dorsal and (B) Ventral views. (C) Lateral view (left side). (D) Oblique lateral, intraorbital view (left). (E) Caudal view. Indications include the rostrum (white brackets), palate and zygomatic arch (white lines), and neurocranium (dashed ovoid). Color indicates landmarks used for calculating the neurocranium centroid (blue).
(TIF)

Figure S2 PC1 GWAS results from PLINK linear regressions with and without a size covariate. (A and B) *x*-axis indicates chromosome, *y*-axis indicates −log_10_(*P*-value). (A) Univariate analysis suggests multiple, highly significant loci are associated with PC1 skull shape. (B) Correction for size using breed averages of the log(neurocranium centroid) indicates that associations on CFA10 (*HMGA2* locus) and CFA15 (*IGF1* locus) are lost upon correction (compare green arrows).
(TIF)

Figure S3 Selective sweeps detected at QTLs on CFA1, 5, and 30. CFA1 (A–C), CFA5 (D–F), CFA30 (G–I). Line graphs plot 10-SNP sliding window averages for log(H_O_ ratios) (A,D,G) or F_ST_ (B,E,H) for each chromosome. Scatterplots depict regional views of SNP values for H_O_ or F_ST_ and include Lowess best fit curves (C,F,I). Color coding corresponds to dolichocephalic breeds (blue), brachycephalic breeds (purple), and F_ST_ treating brachycephalic breeds as a subpopulation (green).
(TIF)

Figure S4 Overexpression activity differs between BMP3 variants. Overexpression utilized human *BMP3* constructs, since the mature peptides of human and dog/wolf BMP3 are identical. (A–C) Whole mount embryos at embryonic stage 24 hpf, anterior to the left. Phenotypes are representative of (A) normal, (B) dorsalized, (C) mildly dorsalized classes following injection of human *BMP3* mRNA into one-cell staged zebrafish embryos. (D) Stacked bar graph summarizing phenotypes observed following wt *BMP3* or *BMP3^F452L^* mRNA injection. The dysmorphic phenotypes classified as “other” included combinations of mild dorsalization, tail curving, occlusion of the yolk extension, and invariably, hypoplasia or necrosis of head structures. Doses listed are in picograms (pg) of mRNA (*x*-axis). The frequencies of phenotypes are indicated by the *y*-axis. Each dose was repeated five or more times. The number of embryos injected is listed above each dose. Injection of *BMP3^F452L^* more potently dorsalizes embryos compared to wt *BMP3* (student's t-test *P*<0.05 for 25–75 pg doses, <0.01 for 100 pg dose).
(TIF)

Figure S5 Y/F→L substitutions differentially affect Tgfßs. (A–C,E–G) Whole mount embryos at embryonic stage 24 hpf (A–C) or 28 hpf (E–G), anterior to the left. Phenotypes are representative of (A,E) normal, (B) dorsalized, (C) mildly dorsalized, (F) ventralized, (G) mildly ventralized classes following injections. (A–D) Embryos injected with mouse *Gdf1*or *Gdf1^F→L^* mRNA. (E–H) Embryos injected with either zebrafish *bmp2b* or *bmp2b^Y→L^* mRNA. (D,H) Stacked bar graphs depicting frequency of observed phenotypes. Number of embryos injected per mRNA concentration appears above columns. While a missense mutation strongly reduces GDF1 dorsalizing activity, a comparable mutation in Bmp2b has little affect on this molecule's ventralizing activity.
(TIF)

Figure S6 Histogram of PC1 breed-sex values. Extreme brachycephalic breeds were defined by their isolation from the main distribution of PC1.
(TIF)

Table S1 Fifty-one landmarks measured by microscribe digitizer. Location of each landmark is described by the right column (compare with Figure S1).
(XLS)

Table S2 Canid skull sources used in morphometric analyses. ID (first column) refers to each collection's numerical identifiers, when assigned. Collection abbreviations correspond to the following: Skulls Unlimited Museum of Osteology (SUMO), Oklahoma City, OK; Smithsonian National Museum of Natural History (SMNH), Washington, DC; California Academy of Science (CAS), San Francisco, CA; and Naturhistorisches Museum der Burgergemeinde Bern (NMBE). Bandar, and Williams are personal collections. PC1 rank and size quartiles are based on breed averages. Size quartiles are based on the distribution of neurocranium centroid sizes where “1” is smallest, “4” is largest.
(XLSX)

Table S3 CanMap genotype profiles used in skull shape GWAS. The genotypes from a total of 576 CanMap dogs (105 breed-sex combinations) were matched with PC1 breed-sex phenotypes. For 75 CanMap breed-sex combinations, PC1 traits were assigned breed-sex averages. The remaining 30 breed-sex assignments were made based on PC1 data that was derived from a single breed-sex representative. Of the 30 breeds with assigned PC1 values based on single skull representatives, 17 of these had PC1 breed-sex averages for the opposite sex. Nineteen CanMap breeds used genotype data from only one sex, as craniometric data for the opposite sex was unavailable. Asterisks indicate CanMap breeds falling within the 1^st^ and 2^nd^ breed-sex log(neurocranium centroid) quartiles that were used in stratified analysis (Figure 2C).
(XLSX)

Table S4 Associated SNPs and their *p*-values. SNPs listed are the top 100 associations (PLINK) or those that remained significant following correction for multiple testing (Bonferroni adjustment, GEMMA). Minor allele frequencies are based on 576 dogs from the CanMap dataset for whom we collected phenotype data. SNPs are ordered by strength of association and analysis type (GEMMA).
(XLSX)

Table S5 F_ST_ intervals. For each non-allometric QTL identified by GWAS, a single marker and an interval are listed. Intervals indicate regions of contiguous or nearly contiguous blocks of sliding windows whose F_ST_ scores ranked within the top 95^th^ percent of the distribution (F_ST = _0.06). The best marker within each interval is also listed. Note that CFAX.105 is not listed, as sliding windows for this QTL did not exceed the 95^th^ percent cutoff.
(XLSX)

Table S6 Haplotypes inferred by fastPHASE using CanMap genotypes. Ninety-three haplotypes with 5 or more chromosomes are listed. Alleles are color-coded relative to Haplotype 55 (Hap55), the most common haplotype identified, with matching alleles colored blue and differences colored red. Ranges in haplotype frequency (or chromosome sum, right-most column) are color-coded from blue to red, to represent low thru high values, respectively. Among extreme brachycephalic breeds, only 6 haplotypes were inferred (a haplotype unique to one Boston Terrier was omitted from the table above): Haplotypes 51, 55, 56, 65, 130, and 134. Commonality among the six haplotypes spans a 190 kb interval, from markers CFA32.8152258-CFA32.8342370. Notably, this interval extends across *BMP3* and *PRKG2*.
(XLS)

Table S7 Genetic variants discovered via alignment of whole-genome sequencing reads to the 190 kb critical interval (CFA32 markers 8152258–8342370) defined by CanMap haplotype sharing among brachycephalic breeds. Positions correspond to the CanFam2 assembly. Genotypes with quality thresholds ≤8 were reset to “0”. Green shading highlights criteria used for filtering variants. A priori knowledge indicated that causal variant(s) at the CFA32 locus must be located within a critical interval defined by allelic agreement between the Bulldog and Pekingese that were used for sequencing. As such, we reduced our critical interval to variants falling between CFA32 markers 8152258–8237937. In addition to meeting this criterion, variants earmarked “TRUE” under the column “Of potential interest” met one or more of the following criteria: 1) location within or 20 bp adjacent to an exon, 2) phastCons4way score ≥0.7, or 3) an association score (omitting Scottish terriers, see Materials and Methods) falling within the smallest 5% of *P*-values. In total, 48 variants met criteria listed above, including the missense mutation of BMP3 at position 8,196,098 (indicated by boldface).
(XLSX)

Table S8 Dogs genotyped to verify the breakpoint at CFA32:8237937. A panel of 32 Pekingese were genotyped by Sanger sequencing using three markers: CFA32: 8196098 (the *BMP3* missense mutation), CFA32:8237937 and CFA32:8296162. The latter two markers occur downstream of the breakpoint that was detected based on disagreement between the Pekingese and Bulldog whose genomes were sequenced.
(XLSX)

Table S9 A survey of the *BMP3* C/A transversion among 842 dogs and wolves. The *BMP3^F452L^* is caused by a C to A transversion at CFA32:8196098. A total of 113 AKC and FCI recognized breeds were genotyped by Sanger sequencing. Breeds and wolves are ordered according to PC1 rank (high = brachycephalic, low = dolichocephalic). Frequencies are given corresponding to derived (“A”) and ancestral (“C”) alleles. With the exception of Scottish Terriers and one Shetland sheepdog, all carriers of the BMP3 missense mutation rank higher than wolves for PC1. Among breeds in our allele frequency survey without morphological information (rows 77–113), six of eight carriers are assumed to be brachycephalic based on breed club descriptions (marked with asterisks). Green shading reflects allele frequencies.
(XLSX)

Table S10 Primers used for sequencing and subcloning, as indicated by the last column. Name (1st column) refers to Ostrander lab primer identifiers. 5′ Tag (3rd column) refers to primer modifications used to aid sequencing or subcloning. Start and end amplicon positions are based on CanFam2 coordinates.
(XLSX)

## References

[1] Sablin MV, Khlopachev GA (2002). The Earliest Ice Age Dogs: Evidence from Eliseevichi 1.. Current Anthropology.

[2] Germonpré M, Sablin MV, Stevens RE, Hedges RE, Hofreiter M (2009). Fossil dogs and wolves from Palaeolithic sites in Belgium, the Ukraine and Russia: osteometry, ancient DNA and stable isotopes.. Journal of Archaeological Science.

[3] Boyko AR, Quignon P, Li L, Schoenebeck JJ, Degenhardt JD (2010). A simple genetic architecture underlies morphological variation in dogs.. PLoS Biol.

[4] Fondon JW, Garner HR (2004). Molecular origins of rapid and continuous morphological evolution.. Proc Natl Acad Sci USA.

[5] Stockard CR, Anderson OD, James WT, Wistar Institute of Anatomy and Biology (1941). The Genetic and Endocrinic Basis for Differences in Form and Behavior.

[6] Haworth KE, Islam I, Breen M, Putt W, Makrinou E (2001). Canine TCOF1; cloning, chromosome assignment and genetic analysis in dogs with different head types.. Mammalian Genome.

[7] Hunemeier T, Salzano FM, Bortolini MC (2009). TCOF1 T/Servariant and brachycephaly in dogs.. Animal Genetics.

[8] Haworth K, Breen M, Binns M, Hopkinson DA, Edwards YH (2001). The canine homeobox gene MSX2: sequence, chromosome assignment and genetic analysis in dogs of different breeds.. Animal Genetics.

[9] Bannasch D, Young A, Myers J, Truvé K, Dickinson P (2010). Localization of canine brachycephaly using an across breed mapping approach.. PLoS ONE.

[10] Quilez J, Short AD, Martínez V, Kennedy LJ, Ollier W (2011). A selective sweep of >8 Mb on chromosome 26 in the Boxer genome.. BMC Genomics.

[11] Klingenberg CP (2011). MorphoJ: an integrated software package for geometric morphometrics.. Molecular Ecology Resources.

[12] The Complete Dog Book (1998). The Complete Dog Book. 19th ed.

[13] Vaysse A, Ratnakumar A, Derrien T, Axelsson E, Pielberg GR (2011). Identification of Genomic Regions Associated with Phenotypic Variation between Dog Breeds using Selection Mapping.. PLoS Genet.

[14] Jones P, Chase K, Martin A, Davern P, Ostrander EA (2008). Single-nucleotide-polymorphism-based association mapping of dog stereotypes.. Genetics.

[15] Purcell S, Neale B, Todd-Brown K, Thomas L, Ferreira MAR (2007). PLINK: a tool set for whole-genome association and population-based linkage analyses.. Am J Hum Genet.

[16] Sutter NB, Bustamante CD, Chase K, Gray MM, Zhao K (2007). A Single IGF1 Allele Is a Major Determinant of Small Size in Dogs.. Science.

[17] Zhou X, Stephens M (2012). Genome-wide Efficient Mixed Model Analysis for Association Studies.. Nature Genetics.

[18] Parker HG, Kukekova AV, Akey DT, Goldstein O, Kirkness EF (2007). Breed relationships facilitate fine-mapping studies: A 7.8-kb deletion cosegregates with Collie eye anomaly across multiple dog breeds.. Genome Research.

[19] Parker HG (2004). Genetic Structure of the Purebred Domestic Dog.. Science.

[20] Akey JM, Zhang G, Zhang K, Jin L, Shriver MD (2002). Interrogating a high-density SNP map for signatures of natural selection.. Genome Research.

[21] Pollinger JP, Bustamante CD, Fledel-Alon A, Schmutz S, Gray MM (2005). Selective sweep mapping of genes with large phenotypic effects.. Genome Research.

[22] Chikuda H, Kugimiya F, Hoshi K, Ikeda T, Ogasawara T (2004). Cyclic GMP-dependent protein kinase II is a molecular switch from proliferation to hypertrophic differentiation of chondrocytes.. Genes & Development.

[23] Daluiski A, Engstrand T, Bahamonde ME, Gamer LW, Agius E (2001). Bone morphogenetic protein-3 is a negative regulator of bone density.. Nature Genetics.

[24] Pfeifer A, Aszódi A, Seidler U, Ruth P, Hofmann F (1996). Intestinal secretory defects and dwarfism in mice lacking cGMP-dependent protein kinase II.. Science.

[25] Sun Y, Zhang Q-J, Zhong J, Wang Y-Q (2010). Characterization and expression of AmphiBMP3/3b gene in amphioxus Branchiostoma japonicum.. Development, Growth & Differentiation.

[26] Takao M, Hino J, Takeshita N, Konno Y, Nishizawa T (1996). Identification of rat bone morphogenetic protein-3b (BMP-3b), a new member of BMP-3.. Biochem Biophys Res Commun.

[27] Kettunen P, Nie X, Kvinnsland IH, Luukko K (2006). Histological development and dynamic expression of Bmp2-6 mRNAs in the embryonic and postnatal mouse cranial base.. Anat Rec.

[28] Adzhubei IA, Schmidt S, Peshkin L, Ramensky VE, Gerasimova A (2010). A method and server for predicting damaging missense mutations.. Nat Methods.

[29] Allendorph GP, Isaacs MJ, Kawakami Y, Izpisua Belmonte JC, Choe S (2007). BMP-3 and BMP-6 structures illuminate the nature of binding specificity with receptors.. Biochemistry.

[30] Gamer LW, Nove J, Levin M, Rosen V (2005). BMP-3 is a novel inhibitor of both activin and BMP-4 signaling in Xenopus embryos.. Developmental Biology.

[31] Kokabu S, Gamer L, Cox K, Lowery J, Tsuji K (2011). BMP3 Suppresses Osteoblast Differentiation of Bone Marrow Stromal Cells via Interaction with Acvr2b.. Mol Endocrinol.

[32] Mueller RL, Huang C, Ho RK (2010). Spatio-temporal regulation of Wnt and retinoic acid signaling by tbx16/spadetail during zebrafish mesoderm differentiation.. BMC Genomics.

[33] Bonnet C, Andrieux J, Beri-Dexheimer M, Leheup B, Boute O (2010). Microdeletion at chromosome 4q21 defines a new emerging syndrome with marked growth restriction, mental retardation and absent or severely delayed speech.. Journal of Medical Genetics.

[34] Wiley D, Amenta N, Alcantara D, Ghosh D, Kil YJ (2005). Evolutionary morphing..

[35] Scheet P, Stephens M (2006). A Fast and Flexible Statistical Model for Large-Scale Population Genotype Data: Applications to Inferring Missing Genotypes and Haplotypic Phase.. The American Journal of Human Genetics.

[36] Ihaka R, Gentleman R (1996). R: A Language for Data Analysis and Graphics.. Journal of Computational and Graphical Statistics.

[37] Li H, Handsaker B, Wysoker A, Fennell T, Ruan J (2009). The Sequence Alignment/Map format and SAMtools.. Bioinformatics.

[38] Wickham H (2009). ggplot2: Elegant Graphics for Data Analysis (Use R). 2nd ed.

[39] Wickham H (2007). Reshaping data with the reshape package.. J Stat Softw.

[40] Siepel A, Bejerano G, Pedersen JS, Hinrichs AS, Hou M (2005). Evolutionarily conserved elements in vertebrate, insect, worm, and yeast genomes.. Genome Research.

[41] Wall NA, Craig EJ, Labosky PA, Kessler DS (2000). Mesendoderm induction and reversal of left-right pattern by mouse Gdf1, a Vg1-related gene.. Developmental Biology.

[42] Nikaido M, Tada M, Saji T, Ueno N (1997). Conservation of BMP signaling in zebrafish mesoderm patterning.. Mechanisms of Development.

[43] Villefranc JA, Amigo J, Lawson ND (2007). Gateway compatible vectors for analysis of gene function in the zebrafish.. Dev Dyn.

[44] Kwan KM, Fujimoto E, Grabher C, Mangum BD, Hardy ME (2007). The Tol2kit: a multisite gateway-based construction kit for Tol2 transposon transgenesis constructs.. Dev Dyn.

[45] Thisse C, Thisse B (2008). High-resolution in situ hybridization to whole-mount zebrafish embryos.. Nat Protoc.

[46] Schilling TF, Piotrowski T, Grandel H, Brand M, Heisenberg CP (1996). Jaw and branchial arch mutants in zebrafish I: branchial arches.. Development.

[47] Neuwirth E (2007). RColorBrewer: ColorBrewer palettes..

[48] Turner S, Bush W (2010). Getting Genetics Done.. http://gettinggeneticsdone.blogspot.com/.

