# Supplementary figures and images for: Variation of BMP3 Contributes to Dog Breed Skull Diversity
Source: PLoS Genet. 2012 Aug 2;8(8):e1002849. doi: 10.1371/journal.pgen.1002849 (PMC3410846; doi:10.1371/journal.pgen.1002849)

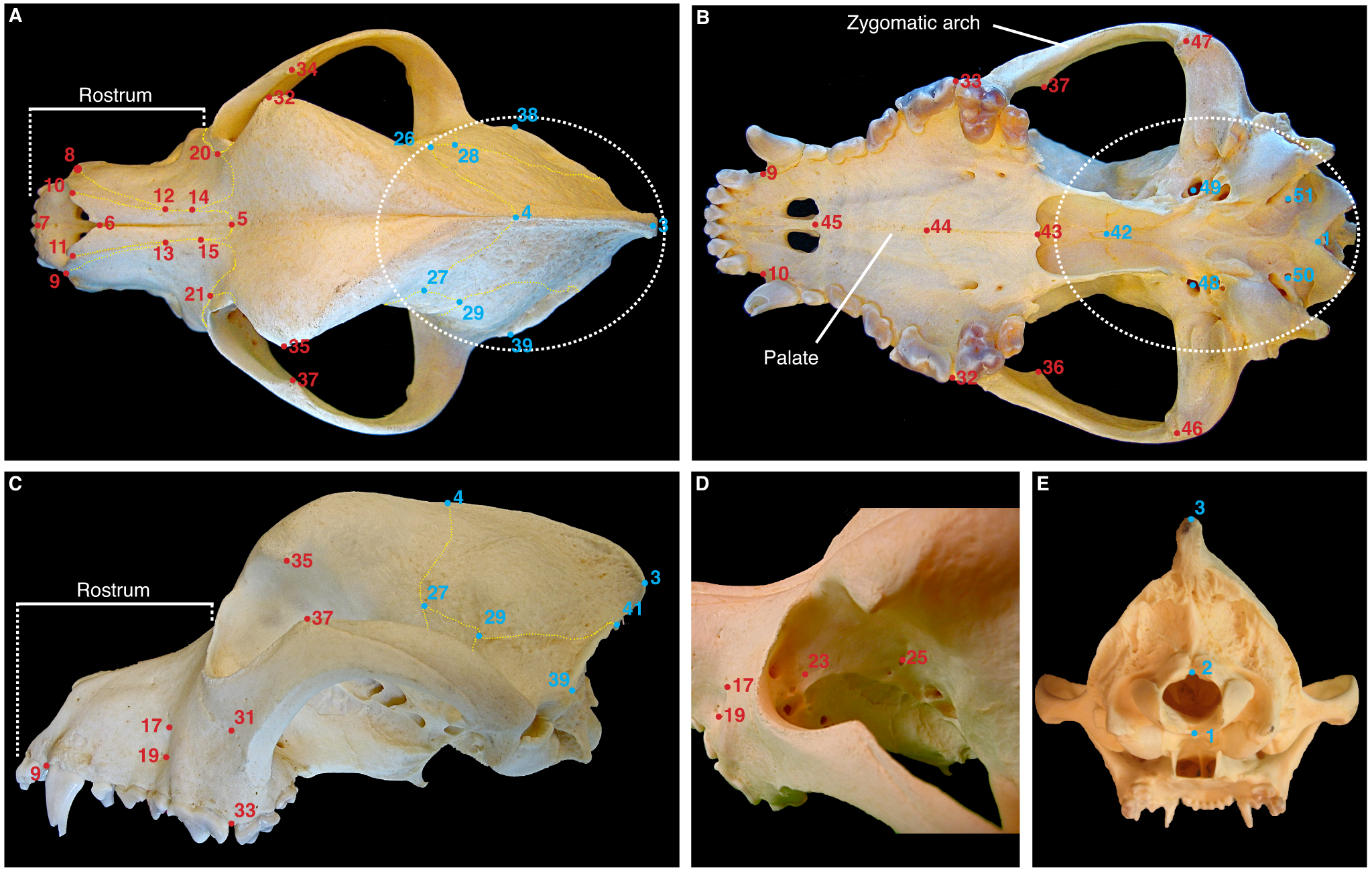

Supplement: Figure S1 — Diagrams of 51 cranioskeletal landmarks captured with a microscribe digitizer. See Table S1 for anatomical descriptions corresponding to numbering. (a–e) Anterior facing left. (A) Dorsal and (B) Ventral views. (C) Lateral view (left side). (D) Oblique lateral, intraorbital view (left). (E) Caudal view. Indications include the rostrum (white brackets), palate and zygomatic arch (white lines), and neurocranium (dashed ovoid). Color indicates landmarks used for calculating the neurocranium centroid (blue). (TIF) [file pgen.1002849.s001.tif]

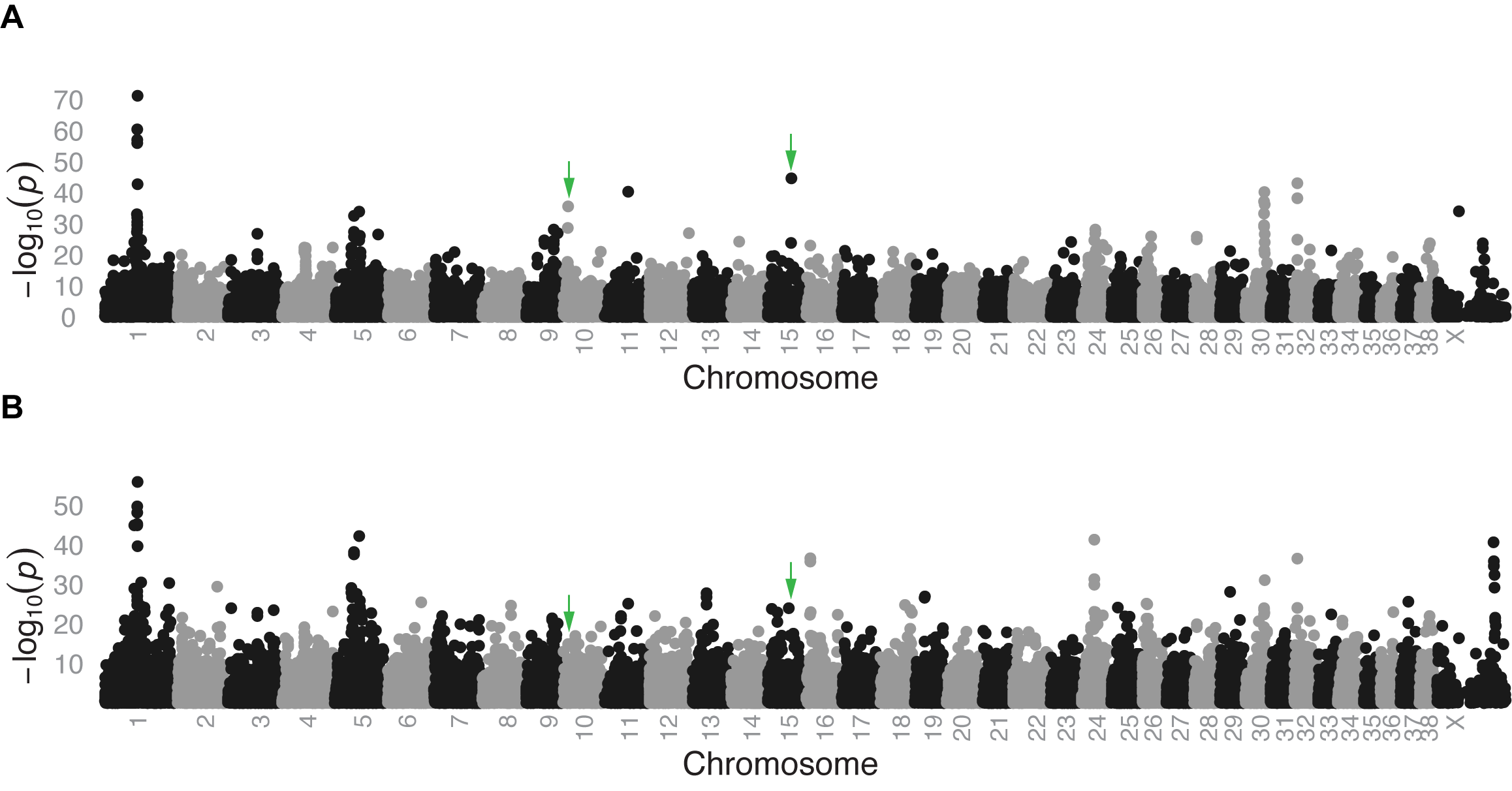

Supplement: Figure S2 — PC1 GWAS results from PLINK linear regressions with and without a size covariate. (A and B) x-axis indicates chromosome, y-axis indicates −log10(P-value). (A) Univariate analysis suggests multiple, highly significant loci are associated with PC1 skull shape. (B) Correction for size using breed averages of the log(neurocranium centroid) indicates that associations on CFA10 (HMGA2 locus) and CFA15 (IGF1 locus) are lost upon correction (compare green arrows). (TIF) [file pgen.1002849.s002.tif]

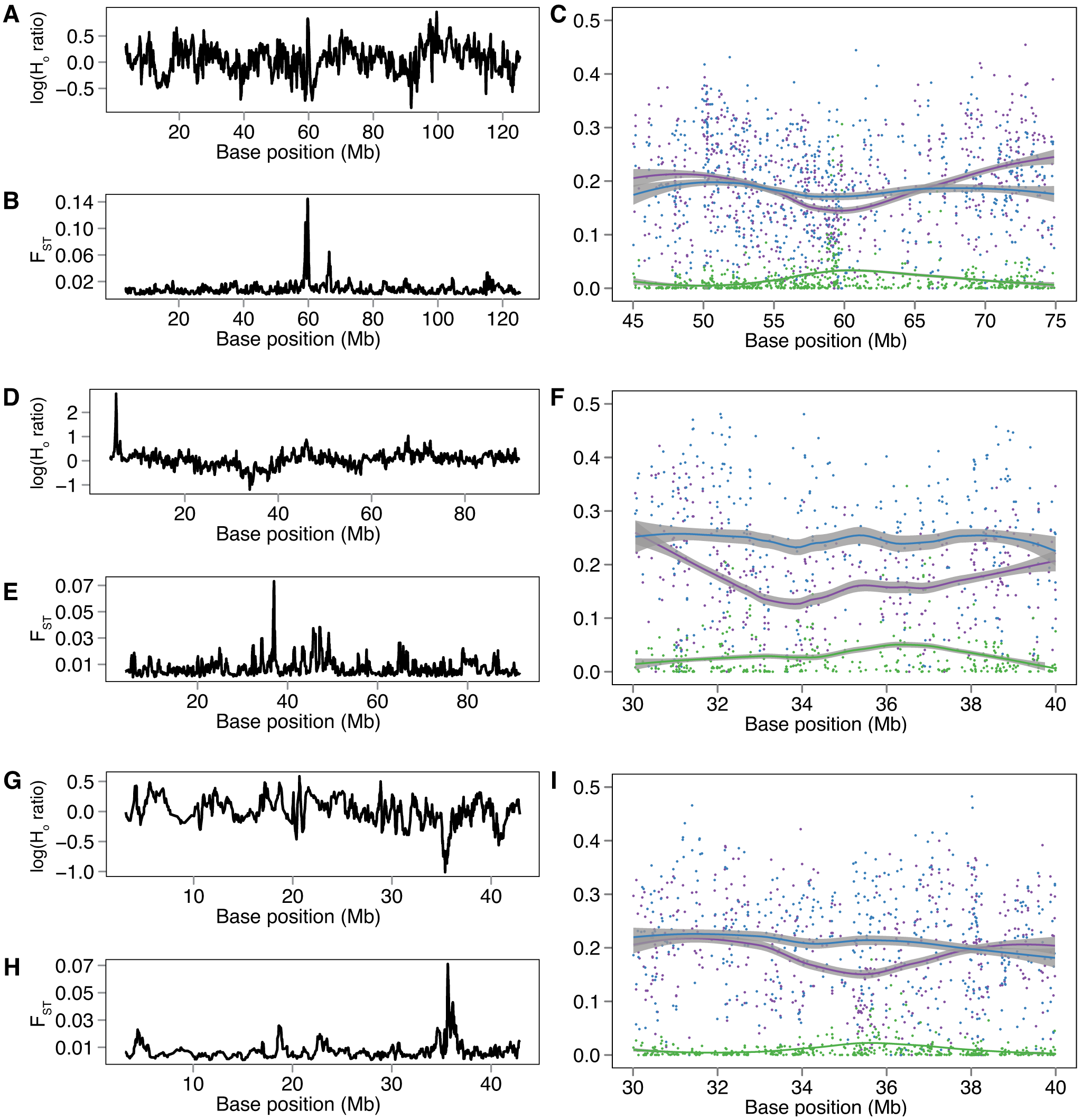

Supplement: Figure S3 — Selective sweeps detected at QTLs on CFA1, 5, and 30. CFA1 (A–C), CFA5 (D–F), CFA30 (G–I). Line graphs plot 10-SNP sliding window averages for log(HO ratios) (A,D,G) or FST (B,E,H) for each chromosome. Scatterplots depict regional views of SNP values for HO or FST and include Lowess best fit curves (C,F,I). Color coding corresponds to dolichocephalic breeds (blue), brachycephalic breeds (purple), and FST treating brachycephalic breeds as a subpopulation (green). (TIF) [file pgen.1002849.s003.tif]

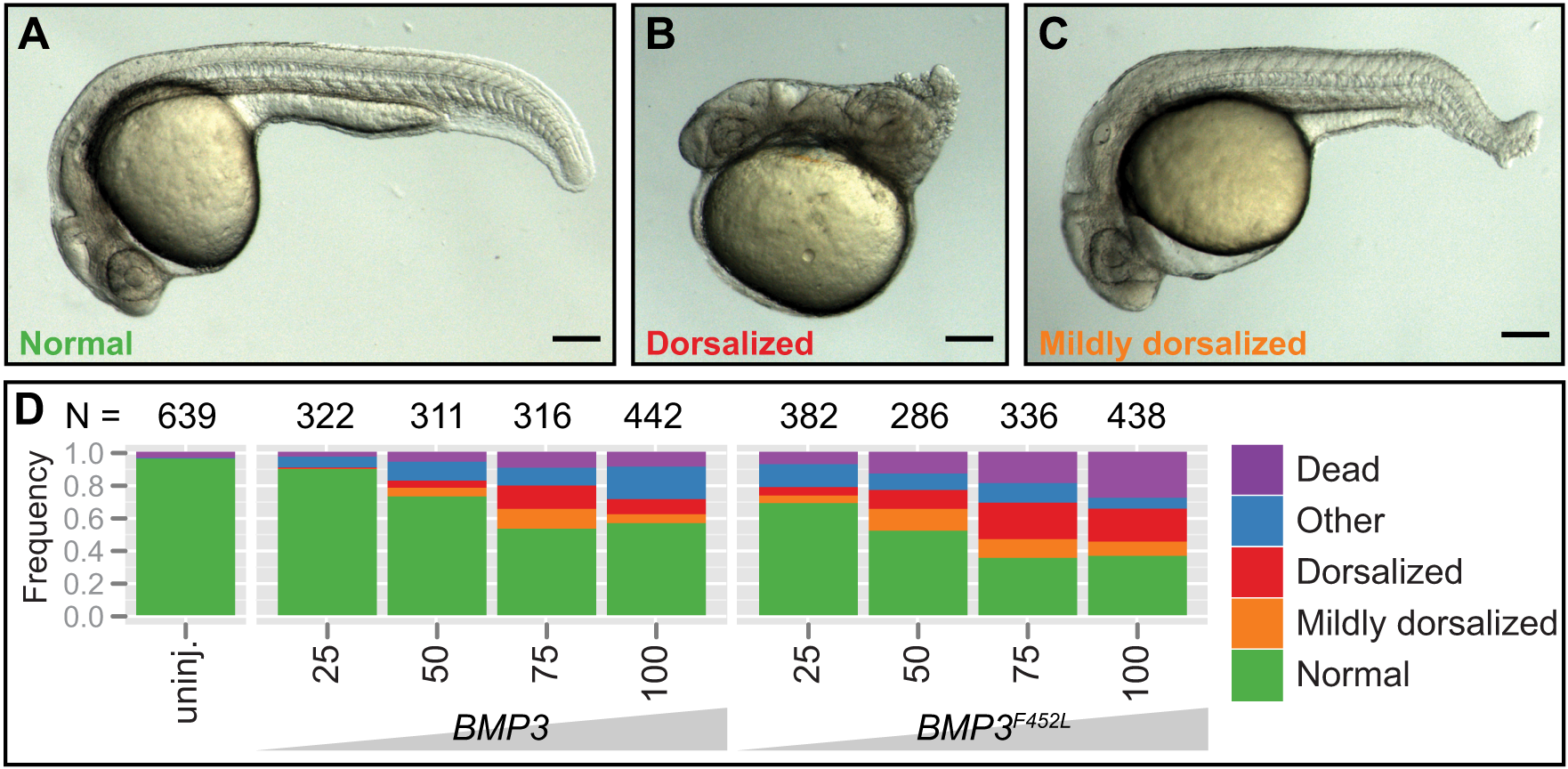

Supplement: Figure S4 — Overexpression activity differs between BMP3 variants. Overexpression utilized human BMP3 constructs, since the mature peptides of human and dog/wolf BMP3 are identical. (A–C) Whole mount embryos at embryonic stage 24 hpf, anterior to the left. Phenotypes are representative of (A) normal, (B) dorsalized, (C) mildly dorsalized classes following injection of human BMP3 mRNA into one-cell staged zebrafish embryos. (D) Stacked bar graph summarizing phenotypes observed following wt BMP3 or BMP3F452L mRNA injection. The dysmorphic phenotypes classified as “other” included combinations of mild dorsalization, tail curving, occlusion of the yolk extension, and invariably, hypoplasia or necrosis of head structures. Doses listed are in picograms (pg) of mRNA (x-axis). The frequencies of phenotypes are indicated by the y-axis. Each dose was repeated five or more times. The number of embryos injected is listed above each dose. Injection of BMP3F452L more potently dorsalizes embryos compared to wt BMP3 (student's t-test P<0.05 for 25–75 pg doses, <0.01 for 100 pg dose). (TIF) [file pgen.1002849.s004.tif]

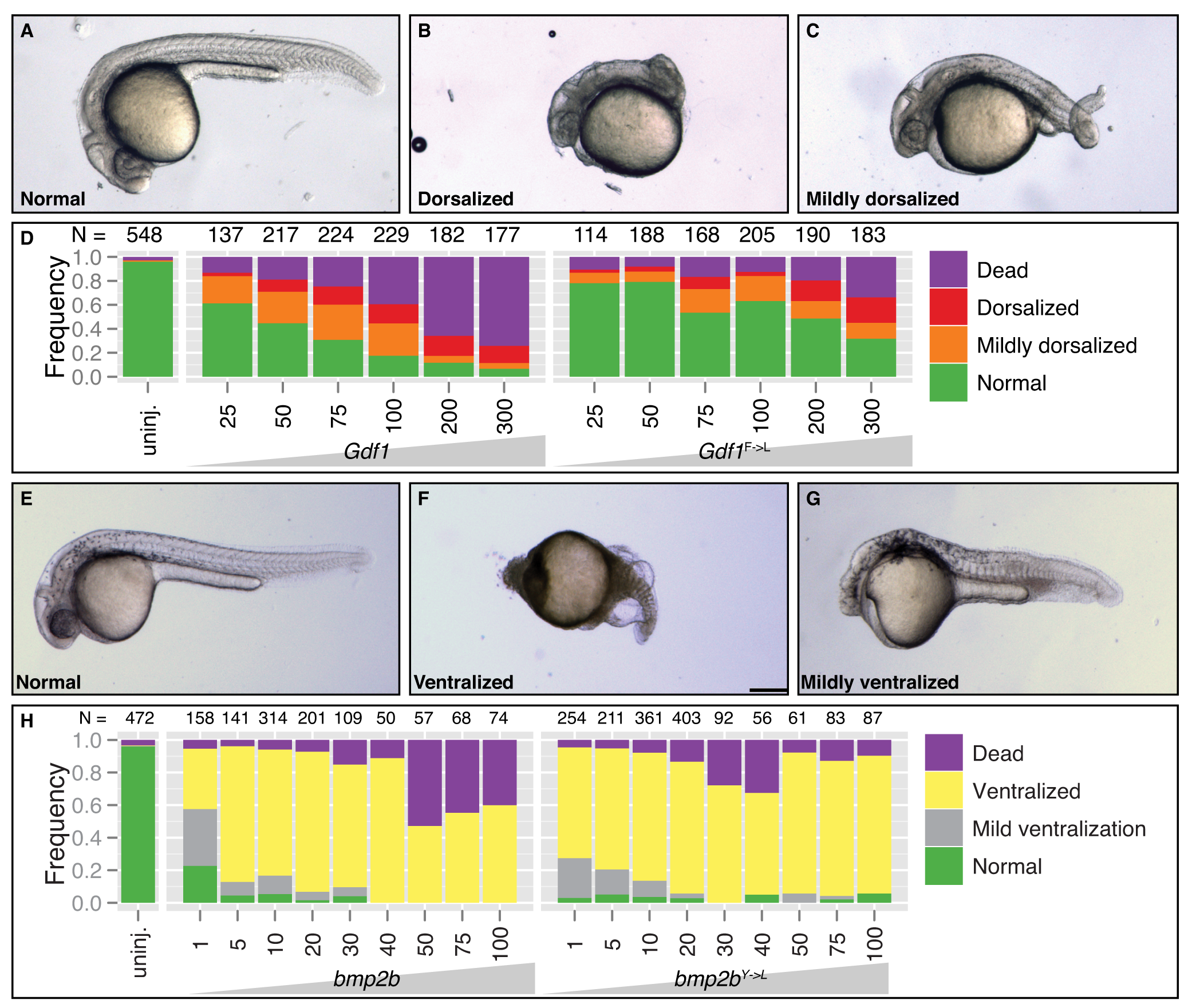

Supplement: Figure S5 — Y/F→L substitutions differentially affect Tgfßs. (A–C,E–G) Whole mount embryos at embryonic stage 24 hpf (A–C) or 28 hpf (E–G), anterior to the left. Phenotypes are representative of (A,E) normal, (B) dorsalized, (C) mildly dorsalized, (F) ventralized, (G) mildly ventralized classes following injections. (A–D) Embryos injected with mouse Gdf1or Gdf1F→L mRNA. (E–H) Embryos injected with either zebrafish bmp2b or bmp2bY→L mRNA. (D,H) Stacked bar graphs depicting frequency of observed phenotypes. Number of embryos injected per mRNA concentration appears above columns. While a missense mutation strongly reduces GDF1 dorsalizing activity, a comparable mutation in Bmp2b has little affect on this molecule's ventralizing activity. (TIF) [file pgen.1002849.s005.tif]

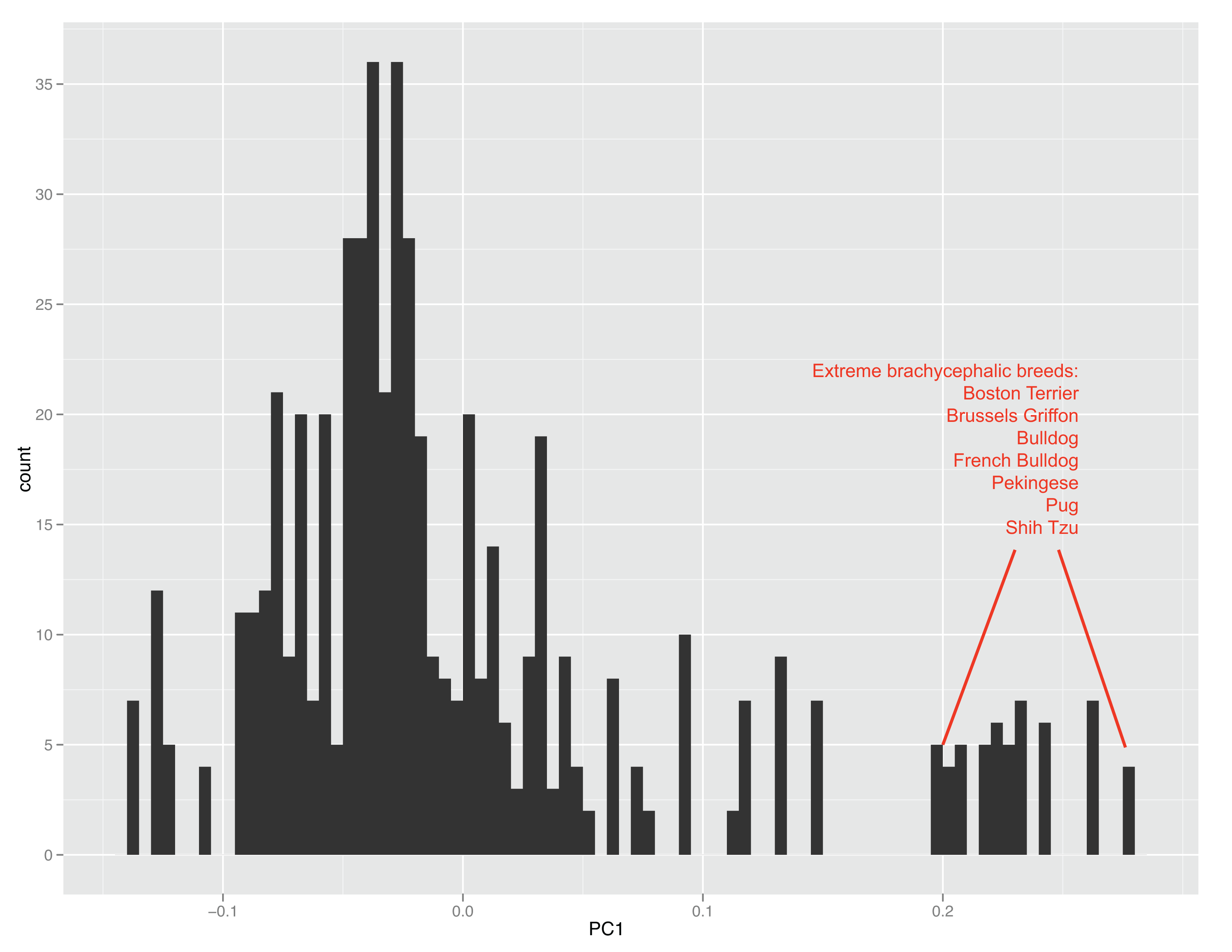

Supplement: Figure S6 — Histogram of PC1 breed-sex values. Extreme brachycephalic breeds were defined by their isolation from the main distribution of PC1. (TIF) [file pgen.1002849.s006.tif]
